# The Sunny South

**Published:** 1876-12

**Authors:** 


*The Sunny South*, a weekly literary paper, published in Atlanta, Georgia, is now regarded as the best exponent of the literary talent in the Southern States. It is tastefully and elegantly gotten up, and is unsurpassed by anything of the kind in the United States. Much of the literature of the present day is light, trashy, and demoralizing. Not so the *Sunny South*, which may safely be admitted into our families, and will instruct, while it gratifies that laudable taste for a refined and elevated literature which we are glad to know exists amongst the intelligent classes of Southern society. It is edited by Mr. J. H. Seals, an able and experienced journalist, assisted by Mrs. Mary E. Bryant, one of the most brilliant and charming female writers of the age.

